# Learning geometric Jensen-Shannon divergence for tiny object detection in remote sensing images

**DOI:** 10.3389/fnbot.2023.1273251

**Published:** 2023-11-09

**Authors:** Shuyan Ni, Cunbao Lin, Haining Wang, Yang Li, Yurong Liao, Na Li

**Affiliations:** ^1^Department of Electronic and Optical Engineering, Space Engineering University, Beijing, China; ^2^Institute of Artificial Intelligence, Beihang University, Beijing, China; ^3^Aerospace Optical-Microwave Integrated Precision Intelligent Sensing, Key Laboratory of Ministry of Industry and Information Technology, Beihang University, Beijing, China

**Keywords:** tiny object detection, remote sensing images, Jensen-Shannon divergence, deep learning, Gaussian distribution

## Abstract

Tiny objects in remote sensing images only have a few pixels, and the detection difficulty is much higher than that of regular objects. General object detectors lack effective extraction of tiny object features, and are sensitive to the Intersection-over-Union (IoU) calculation and the threshold setting in the prediction stage. Therefore, it is particularly important to design a tiny-object-specific detector that can avoid the above problems. This article proposes the network JSDNet by learning the geometric Jensen-Shannon (JS) divergence representation between Gaussian distributions. First, the Swin Transformer model is integrated into the feature extraction stage as the backbone to improve the feature extraction capability of JSDNet for tiny objects. Second, the anchor box and ground-truth are modeled as two two-dimensional (2D) Gaussian distributions, so that the tiny object is represented as a statistical distribution model. Then, in view of the sensitivity problem faced by the IoU calculation for tiny objects, the JSDM module is designed as a regression sub-network, and the geometric JS divergence between two Gaussian distributions is derived from the perspective of information geometry to guide the regression prediction of anchor boxes. Experiments on the AI-TOD and DOTA datasets show that JSDNet can achieve superior detection performance for tiny objects compared to state-of-the-art general object detectors.

## 1. Introduction

With the rapid development of satellite technology, remote sensing images collected by optical payloads often have a large width and high resolution, so the remote sensing images often contain a large number of tiny objects, which makes remote sensing object detection challenging in applications such as maritime search and rescue, flight scheduling, and ground surveillance. Despite the significant success of deep learning and convolutional neural networks (CNNs), many object detectors can perform various visual detection tasks with high quality (Liu et al., 2016, 2020; Ren et al., 2017; Bochkovskiy et al., 2020), such as salient object detection and crowd density detection. Since the object size and distribution of these scenes are very different with remote sensing images, it is particularly important to design a tiny object detection method suitable for remote sensing scenes.

**Figure 1:**
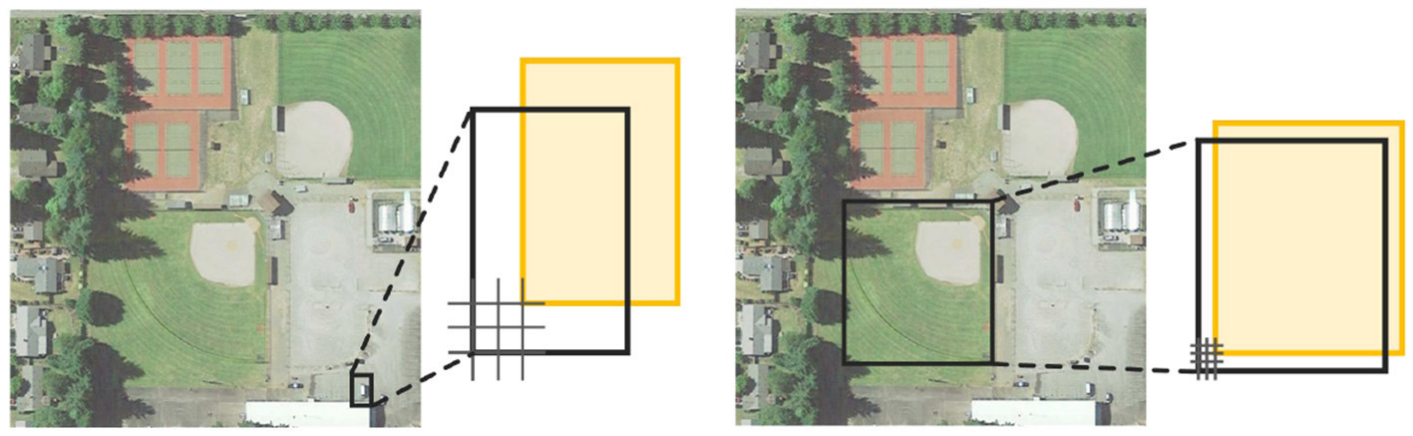
The area difference of the IoU calculation with objects of different pixel sizes. It shows that the IoU calculation method is sensitive to tiny remote-sensing objects.

The latest research in tiny object detection has mainly focused on multi-scale feature learning (Zhang X. et al., 2022), context-based detection (Zhang K. et al., 2022), network structure-based optimization (Lu et al., 2023), data augmentation strategies (Kim and Hwang, 2022) and so on. The above methods are all implemented by the CNN architecture, which drives the data training by enhancing the tiny object feature representation. In addition to the representation of tiny object features, the computation of the IoU for network training is also affected by object scale changes (Li et al., 2021). The IoU between the ground-truth and anchor box directly reflects the positive and negative categories of the current anchor box, but the IoU is sensitive to objects of different pixel sizes, and a small position offset leads to a large change in the IoU value. As shown in Figure 1, the results of the IoU calculation for objects with different pixel sizes are different. For example, when the offset pixel is 2, the IoU value of the object of 8 × 8 pixels is calculated as 0.39, and the IoU value of the object of 96 × 96 pixels is calculated as 0.92. Thus, the results are different. Then, when the IoU threshold is used to determine the positive and negative sample labels of the current anchor box, there is inaccurate classification of the respective labels. Therefore, the IoU calculation method is not suitable for the anchor-box label assignment mechanism of tiny objects. In addition, when there is no overlap or mutual inclusion between the anchor boxes and the ground-truth, the value of IoU remains unchanged and cannot reflect the positional regression relationship between the current anchor box and the ground-truth. This is often the case with tiny objects in remote sensing image.

Based on the application potential of the Swin Transformer model (Liu et al., 2021) in the remote sensing field, this article proposes a new object metric representation learning method (JSDNet), which uses the geometric JS divergence to measure the distribution distance of anchor boxes and the ground-truth. The closed form of geometric JS divergence was previously used to train Bayesian neural networks in reference (Deasy et al., 2020; Thiagarajan and Ghosh, 2022), which brings new inspiration to train deep networks. In this article, feature extraction based on Swin Transformer is firstly performed to find deeper feature representations and richer semantic information. Second, an object regression module (JSDM) is designed to model the object bounding box as a 2D Gaussian distribution, and we use the proposed geometric JS divergence with a closed-form to measure the similarity between the anchor boxes and ground-truth, avoiding the traditional IoU calculation which results in pixel offset sensitivity for tiny objects. The experiments on AI-TOD (Xu et al., 2022) and DOTA (Xia et al., 2018) datasets demonstrate the advanced performance of the proposed method.

We summarize the main contributions as follows:

1. Swin Transformer is integrated into the CNN architecture, and a remote sensing tiny object detector called JSDNet is proposed. The semantic features of the object are extracted by Swin Transformer, and the CNN network is used for the classification and regression processes.
2. The geometric JS divergence with a closed-form is used as the distance measure between Gaussian distributions, which guides the regression loss branch of the object detection network, avoids the sensitivity of the IoU calculation method to tiny objects, and improves the detection performance of remote sensing tiny objects.
3. The regression loss is described from the perspective of information geometry, which provides a new thinking for the algorithm improvement in the tiny object detection field.

## 2. Related works

### 2.1. Tiny object detection

Currently, research on tiny object detection mainly focuses on anchor-based optimization, network structure-based optimization, multi-scale feature learning, context-based information, and label classification strategy.

#### 2.1.1. Anchor-based optimization

Anchors are multiple bounding boxes with different sizes and aspect ratios that are generated centered on each pixel of the image. Yang et al. (2018) propose a dynamic mechanism named MetaAnchor, which can select appropriate anchor for dynamic generation. Zhang et al. (2017) propose a scale compensation anchor matching mechanism to improve the recall rate for tiny objects. Duan et al. (2019) propose using center points to improve the discrimination and screening ability of anchor. Tian et al. (2019) solved the problem of hyperparametric calculation caused by too many anchors. Yang et al. (2019) use point sets to represent the bounding box of tiny objects. Due to the large and dense number of tiny objects in the image, the effectiveness of current tiny object detection algorithms based on anchor still needs to be improved.

#### 2.1.2. Network structure-based optimization

Optimizing backbone and neck can generally enhance feature extraction for objects and improve the performance of tiny object detection (Bochkovskiy et al., 2020). Qiao et al. (2021) designed a recursive feature pyramid as a backbone network. Kong et al. (2020) designed new detection heads that can directly learn the possibility of tiny objects. Cai and Vasconcelos (2018) proposed a multi-stage network structure to improve the value of IoU layer by layer, solving the problem of over fitting in training.

#### 2.1.3. Multi-scale feature learning

Shallow networks contain coordinate information of tiny objects, and using multi-scale feature learning can better fuse and enhance the features of tiny objects. Liu et al. (2016) proposed a single shot multi box detector (SSD) algorithm for hierarchical detection of feature maps of different scales. Lu et al. (2019) designed grid points for spatial feature information fusion. Han et al. (2022) proposed a multi-scale residual block, which obtains multi-scale context information by using dilated convolution in cascaded residual blocks. Literature (Deng et al., 2022; Zeng et al., 2022) improves the feature pyramid network, which can effectively solve the problem that feature coupling at different scales affects the performance of tiny object detection.

#### 2.1.4. Context-based information

Effectively utilizing the background environment information around tiny objects can effectively improve the performance of tiny object detection. Feng et al. (2021) introduced the global context aware enhancement module, which activates the characteristics of the entire object by capturing the global visual context. Li et al. (2019), Leng et al. (2021), Cui et al. (2022) improved the performance of tiny object detection by constructing high-resolution and strong semantic feature maps.

#### 2.1.5. Label classification strategy

Assigning high-quality anchor boxes to tiny objects is challenging, and many recent work has been carried out (Ge et al., 2021). Kim and Lee (2020) proposed probabilistic anchor assignment, which assumes that the joint loss distribution of positive and negative samples follows a Gaussian distribution. Xu et al. (2022) proposed a ranking-based allocation strategy, significantly improving the impact of label allocation on tiny object detection.

### 2.2. 2D Gaussian modeling for remote sensing object

IoU guided regression losses in object detection may lead to deviations in numerical calculations due to the following two issues: The loss form is not differentiable, and the loss calculated by IoU is inconsistent with the assessment. In order to solve the above challenges in remote sensing images, Yang et al. (2021a,b, 2023) proposed to represent an oriented object as a two-dimensional Gaussian distribution of rotation, which brought new inspiration for object detection. Modeling a remote sensing object as a 2D Gaussian distribution *N*(*m*, Σ) at any angle:


(1) $$\left\{ \begin{array}{l} m=(x,y{)}^{T}\\ \Sigma^{1/2}=R\Lambda R^{T} \end{array}\right.$$

where, *R* represents a 2D rotation matrix and Λ represents a diagonal matrix of eigenvalues. Specifically, the anchor box and the ground-truth of the object are modeled as two 2D rotational Gaussian distributions, and then the distance between the two Gaussian distributions is measured to guide the regression network in training. The design of regression loss function can effectively adapt to the situation of orienting and dense object distribution in remote sensing image. Yang et al. (2021a) used Wasserstein distance for spatial distance measurement, while Yang et al. (2021b) used Kullback-Leibler divergence. These metrics are not closed forms in information geometry field.

## 3. Proposed method

### 3.1. Overall framework

**Figure 2:**
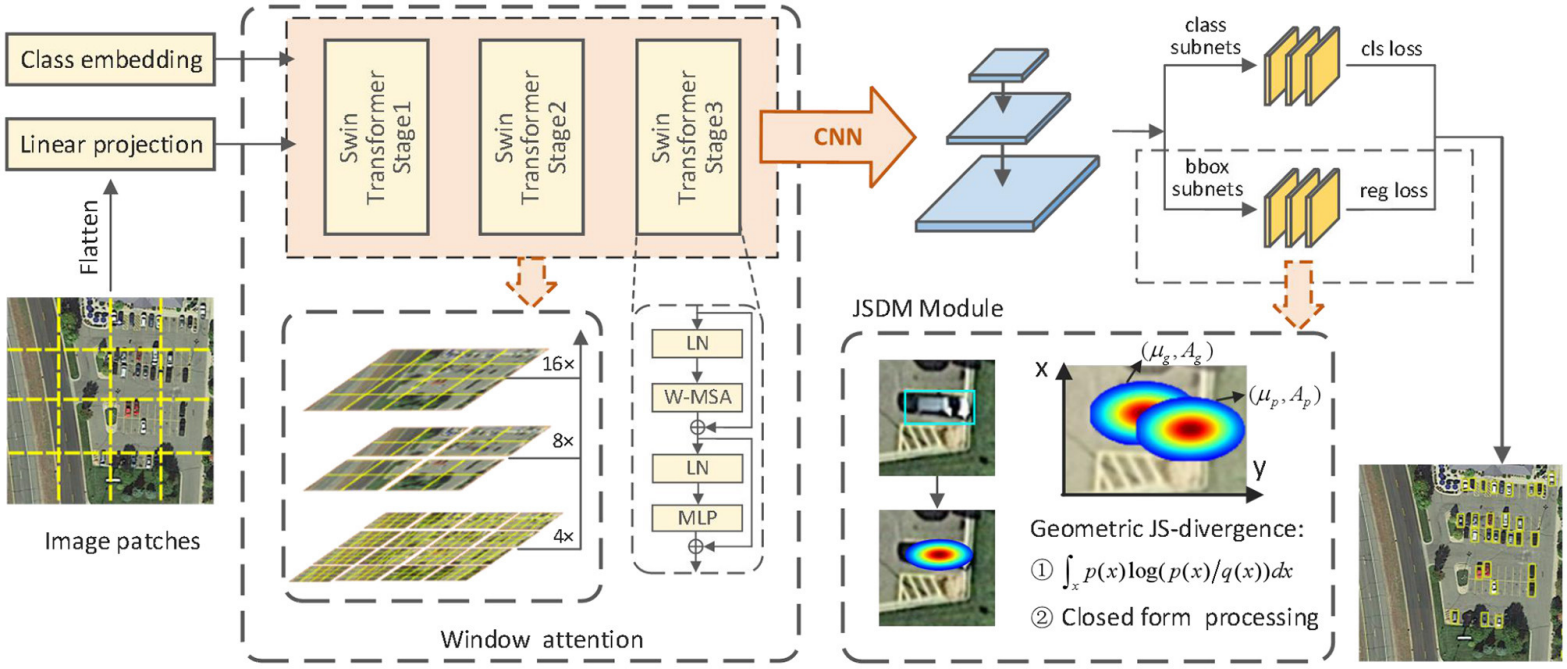
The proposed JSDNet framework. It consists of the Swin Transformer backbone, feature fusion module, and classification and regression sub-networks. The regression sub-network JSDM uses the geometric JS divergence for closed-form processing.

The proposed tiny-object-detection framework JSDNet is shown in Figure 2, using RetinaNet (Lin et al., 2020) as the baseline algorithm. The framework comprises three main parts: the window attention backbone, the feature fusion network and the detection sub-network. First, Swin Transformer is used as the backbone for feature extraction. Owing to the large width and high pixel characteristics of remote sensing images, the original backbone of RetinaNet cannot effectively extract fine small object features from remote sensing images. Therefore, it is theoretically valid to use window-based self-attention operations. Swin Transformer processes the image into patches, proposes the concept of a moving window, and only calculates self-attention inside the window, which can effectively reduce the length of the sequence and reduce the computational complexity. JSDNet uses Swin Transformer as the backbone, which can handle the problem of different scale features hierarchically and then optimize the detection of remote sensing tiny objects by multi-scale feature maps. Second, JSDNet inputs the obtained multi-scale feature map into the feature pyramid network for feature fusion. The fusion process adopts a top-down transfer method to transfer the high-level feature semantics to the underlying structure. This is the same as the original feature fusion structure of RetinaNet.

Then, JSDNet feeds the fused features into the detection sub-network, which performs label classification and bounding-box regression tasks. In the bounding-box regression task, the JSDM models the object as a 2D Gaussian distribution from information geometry perspective and uses the abstract mean to calculate the geometric JS divergence, so that the JS divergence can be approximated as a similarity measurement of two Gaussian distributions that can produce closed-form expressions.

### 3.2. Gaussian distribution modeling for bounding box

Yang et al. (2021a,b, 2023) proposed that the oriented object is represented as a rotating 2D Gaussian distribution, which brings new inspiration for object detection. However, a tiny object has a small number of pixels in the image, and the IoU calculation method is easily affected by the threshold setting. Modeling the tiny object as a 2D Gaussian distribution can avoid this problem and can also distinguish the object information from the redundant background. Specifically, the anchor box and ground-truth are represented by four parameters (*x*_0_, *y*_0_, *w, h*) of a centroid notation, where (*x*_0_, *y*_0_) represents the coordinates of the rectangle center point, *w* and *h* are the length and width of the rectangle, respectively. At this time, it is described as an inscribed ellipse as follows:


(2) $$\begin{array}{l} \frac{4{\left(x-x_{0}\right)}^{2}}{w^{2}}+\frac{4{\left(y-y_{0}\right)}^{2}}{h^{2}}=1 \end{array}$$

where $\frac{w}{2}$ and $\frac{h}{2}$ are semi-major axes of the ellipse, which are equivalent to half the length and width of the rectangle, respectively.

According to probability statistics, the probability density function of the 2D Gaussian distribution is as follows:


(3) $$f(\text{x | }\mu ,\sum )=\frac{\exp(-\frac{1}{2}{\left(x-\mu \right)}^{\text{T}}\overset{-1}{\sum }(x-\mu ))}{2\pi |\sum {|}^{\frac{1}{2}}}$$

where **x** denotes the coordinate variable (*x, y*), **μ** denotes the mean vector, and ∑ denotes the covariance matrix. When the inscribed ellipse in (1) is set as a standard 2D Gaussian distribution, there is a conversion relationship between the elliptic and the Gaussian distribution in (3):


(4) $$\mu =\left[\begin{array}{c} x_{0}\\ y_{0} \end{array}\right]\;,\;\sum =\left[\begin{array}{cc} \frac{w^{2}}{4} & 0\\ 0 & \frac{h^{2}}{4} \end{array}\right]$$

At this time, both the ground-truth and anchor box can be modeled as a 2D Gaussian distribution according to the above-mentioned corresponding relationship.

### 3.3. Closed-form metrics for geometric JS divergence

Let (χ, *F*) be the measurable space of the image plane, χ be the sample space, and *F* be the σ−*algebra* of the measurable events. Denote the distribution variable established in last section as a positive measure μ, the predicted frame of the object as *P*(μ_1_, Σ_1_), and the true frame of the object as *G*(μ_2_, Σ_2_). At this time, the most basic distribution distance KL Divergence can be defined as follows:


(5) $$\begin{array}{l} KL\left(P:G\right):=KL*\left(G:P\right)=\int p\log\left(p/g\right)d\mu \end{array}$$

where *p* and *g* represent the Radon-Nikodym derivatives of the Gaussian distribution *P* and *G* for the positive measure μ, respectively, and “*” represents the inverse distance. It is clear that the KL divergence is an asymmetric distance. One method to achieve symmetric KL divergence is to convert to standard JS divergence, as follows:


(6) $$\begin{array}{l} JS\left(P:G\right):=\frac{1}{2}\left(KL\left(P:\frac{P+G}{2}\right)+KL\left(G:\frac{P+G}{2}\right)\right) \end{array}$$

Yang et al. (2021b) discussed the use of JS divergence for distance measurement. However, the direct application of the above JS divergence to the distance metric leads to a problem where we ignore that the JS divergence between two Gaussian distributions is not available in closed form. Thus, we can hardly obtain a strict distance metric result and thus cannot accurately guide the regression process of the anchor box. Therefore, the JS divergence calculation for remote sensing tiny objects needs to use the closed-form formula, and the closed form can be obtained according to the given exponential family.

**Definition 1** (Abstract mean function, AM). The abstract mean function *AM*(., .) is a continuous binary function, and on the domain of definition *S* ⊂ ℝ_+_, it satisfies the bounded range as follows:


(7) $$\begin{array}{l} \inf\left\{x,y\right\}\leq AM\left(x,y\right)\leq \sup\left\{x,y\right\},\;\forall x,y\in S \end{array}$$

According to Frank (2019), based on *AM*, we construct a weighted expression *AM*_α_(*p, g*) for probability distributions with densities *p* and *g*, where *α* ∈ [0, 1].

**Definition 2** (Geometric statistical mixture, GSM). For the abstract mean function *AM*_α_(*p, g*), with probability densities *p* and *g*, the mixture of distributions *P* and *G* with respect to the geometric mean M can be defined as:


(8) $$\begin{array}{l} {\left(PG\right)}_{\alpha }^{M}(\mu ):=\frac{AM_{\alpha }(P(\mu ),G(\mu ))}{N_{\alpha }^{M}\left(P:G\right)}\\ \;=\exp\left((1\text{​}-\text{​}\alpha )P\text{​}+\text{​}\alpha G\text{​}-\text{​}\log N_{\alpha }^{M}\left(P\text{​}:\text{​}G\right)\right) \end{array}$$

where $N_{\alpha }^{M}\left(:\right)$ is the normalization sub-function. Now, for the distributions *P* and *G*, a statistical mixture function weighted by the geometric is obtained.

**Definition 3** (Mean JS-divergence, AM-JS-divergence). Extending the concept of a geometric statistical mixture to the JS-divergence of two exponential family distributions, we obtain a generalized weighted form of geometric JS-divergence, and it is geometrically symmetric. The definition of mean JS divergence is as follows:


(9) $$\begin{array}{l} {\left(JS\right)}^{M}:=\left(1-\alpha \right)KL\left(P:\;{\left(PG\right)}_{\alpha }^{M}\right)+\alpha KL\left(G:\;{\left(PG\right)}_{\alpha }^{M}\right) \end{array}$$

In particular, when α = 0 or α = 1, no significant mean JS divergence is obtained. The weights α imply a geometrical statistical mixture, so when *α* ∈ ∀(0, 1), (*JS*)^*M*^ can be used as the generalized JS divergence of the two exponential family distributions *P* and *G*.

**Proposition** Assuming that the prediction box and ground-truth in the image conform to the 2D Gaussian distribution in the exponential family distribution and are denoted as *P*(μ_1_, Σ_1_) and *G*(μ_2_, Σ_2_), respectively, the geometric mean JS divergence between them can be expressed as follows:


(10) $$\begin{array}{l} \;{\left(JS\right)}^{G_{\alpha }}(p(\mu_{1},\Sigma_{1}):p(\mu_{2},\Sigma_{2}))\\ =\frac{1}{2}(tr\left(\Sigma_{\alpha }^{-1}((1\text{​}-\text{​}\alpha )\Sigma_{1}\text{​}+\text{​}\alpha \Sigma_{2})\right)+\log\frac{\left|\Sigma_{\alpha }\right|}{{\left|\Sigma_{1}\right|}^{1-\alpha }{\left|\Sigma_{2}\right|}^{\alpha }}-\text{​}2+\\ (1\text{​}-\text{​}\alpha )(\mu_{\alpha }\text{​}-\text{​}\mu_{1}{)}^{\text{T}}\Sigma_{\alpha }^{-1}(\mu_{\alpha }\text{​}-\text{​}\mu_{1})\text{​}+\text{​}\alpha (\mu_{\alpha }\text{​}-\text{​}\mu_{2}{)}^{\text{T}}\Sigma_{\alpha }^{-1}(\mu_{\alpha }\text{​}-\text{​}\mu_{2})) \end{array}$$

where (μ_α_, Σ_α_) is the center of gravity of the matrix harmonics:


(11) $$\begin{array}{l} \mu_{\alpha }=(\mu_{1}\mu_{2}{)}_{\alpha }^{\mu }=\Sigma_{\alpha }\left((1-\alpha )\Sigma_{1}^{-1}\mu_{1}+\alpha \Sigma_{2}^{-1}\mu_{2}\right),\\ \Sigma_{\alpha }=(\Sigma_{1}\Sigma_{2}{)}_{\alpha }^{\Sigma }={\left((1-\alpha )\Sigma_{1}^{-1}+\alpha \Sigma_{2}^{-1}\right)}^{-1} \end{array}$$

According to proposition, JSDNet can learn the JS divergence representation between the prediction box and ground-truth, and then as the regression process of the anchor box (*x*_0_, *y*_0_, *w, h*). Specifically, the anchor-box regression process is realized by calculating the offset, which is the same as the fine-tuning mechanism of the parameter change of RetinaNet.

**Figure 3:**
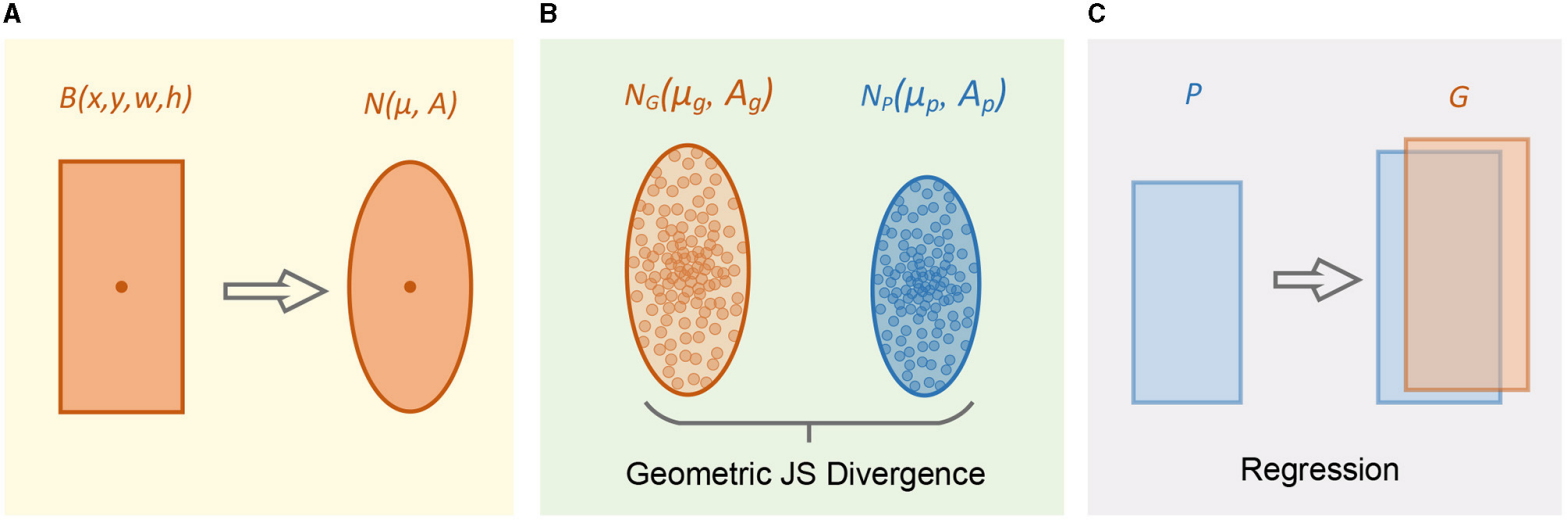
2D space approximation process of geometric mean JS divergence module. **(A)** Modeling a Gaussian distribution for bounding box. **(B)** Calculate geometric JS divergence in 2D space. **(C)** Adjust predicted box to approximate two distributions.

Figure 3 shows the two-dimensional spatial regression calculation process of the JSDM module. First, the bounding box of a tiny object is modeled to obtain a 2D ellipse. Then, the geometric mean JS divergence is used as distance measure between two 2D Gaussian distributions. Finally, update the four parameters of the prediction box to make the regression network converge.

### 3.4. JSDNet training

This section defines the classification and regression loss function for JSDNet. First, a nonlinear relationship between the distance function and the geometric mean JS divergence is established. Specifically, square the geometric mean JS divergence in the proposition and convert it into a fractional form, as follows:


(12) $$\begin{array}{l} {\left(JS\right)}^{G_{\alpha }}\left(N_{P},N_{G}\right)=\frac{1}{\tau +f\left[{\left(JS\right)}^{G_{\alpha }}\left(N_{P}:N_{G}\right)\right]}\;,\;\tau \geq 1 \end{array}$$

where, τ is the offset hyperparameter, *f*(.) is the square operation of the distance function, and belongs to a nonlinear expression. Tiny objects in remote sensing usually occupy a small proportion of pixels, so horizontal bounding boxes are chosen to locate tiny objects. Assuming that the predicted bounding box of tiny objects follows a Gaussian distribution *N*_*P*_ and the ground-truth follows a Gaussian distribution *N*_*G*_, each horizontal bounding box uses a four parameter definition method (*x*_0_, *y*_0_, *w, h*) to represent the center point coordinates and side length of the rectangle. Therefore, the calculation relationship between the relative translation (δ_*x*_, δ_*y*_) and the size scaling (δ_*w*_, δ_*h*_) is as follows, which can guide the horizontal bounding box of tiny objects to update coordinates.


(13) $$\begin{array}{l} \delta_{x}=(G_{x}-x_{a})/w_{a},\;\delta_{y}=(G_{y}-y_{a})/h_{a}\\ \delta_{w}=\log(G_{w}/w_{a}),\;\delta_{h}=\log(G_{h}/h_{a}) \end{array}$$

where, (*x*_*a*_, *y*_*a*_, *w*_*a*_, *h*_*a*_) represents an anchor box for the regression process. The differential calculation of the anchor box regression process may result in a very small value for (13). This typically results in regression losses that are much smaller than classification losses. Therefore, normalizing the mean and variance of (δ_*x*_, δ_*y*_, δ_*w*_, δ_*h*_), and incorporating the geometric mean form JS divergence in the previous section into the standard regression loss function, as shown in Equation (14), can avoid the limitations of traditional IoU loss.


(14) $$\begin{array}{l} L_{reg}=1-{\left(JS\right)}^{G_{\alpha }}\left(N_{P}:N_{G}\right) \end{array}$$

## 4. Experiments

Our experiments are conducted on the AI-TOD and DOTA1.0 datasets and compared with advanced general object detectors to verify the effectiveness of the proposed method for remote sensing tiny object detection.

### 4.1. Experimental settings

(1) Dataset: AI-TOD dataset is a remote sensing tiny object dataset with 28,036 images of 800 × 800 pixels, including eight categories and 700,621 tiny objects. These instances are different from objects in other datasets, as the instances have a small number of pixels. Therefore, the dataset is suitable for training and testing the tiny object detector proposed in this article. We abbreviate the AI-TOD object classes as airplane (APL), bridge (BR), storage-tank (ST), ship (SH), swimming-pool (SP), vehicle (VE), person (PE), and wind-mill (WM). The DOTA1.0 dataset is a public large-scale remote sensing image object detection dataset, with 2,806 satellite or aerial images of about 4,000 × 4,000 pixels, including 15 object categories and 188,282 instances. We only use data augmentation on the DOTA1.0 dataset to avoid network training overfitting.

(2) Evaluation Metrics: We use average precision (AP) and mean average precision (mAP) to compare the performance of different detectors. Also, we refer to the evaluation indicators definition in AI-TOD dataset, including *AP* calculation under different IoU thresholds, and the evaluation of different scales of pixels (*AP*_*vt*_, *AP*_*t*_, *AP*_*s*_ and *AP*_*m*_ represent 2-8 pixels, 8-16 pixels, 16-32 pixels, and 32-64 pixels, respectively), along with the accuracy calculations for each category.

(3) Details: All experiments are performed on a workstation with an NVIDIA RTX 3090 GPU (24G). We use Swin Transformer as the pretrained model for network fine-tuning. During model training, the SGD optimizer is used for gradient descent and updates, the initial learning rate is set to 0.001, and the weight coefficient α are compared with multiple sets of values. The strides for training AI-TOD and DOTA datasets are 320K and 360K, respectively; the weight momentum and decay are set to 0.9 and 0.0001, respectively; and the batch size for training each model is set to 4.

### 4.2. Ablation studies

To verify the effectiveness of the proposed method composition structure, we conduct ablation analysis on two datasets. Table 1 shows the results of using *AP*_50_ ablation to analyze the effect of each component in JSDNet, including the effect of the Transformer structure integrated into the CNN network, the effect of directly using the original JS divergence formula, and the improved effect of using the geometric JS divergence. The comparison shows that the model based on the Transformer backbone can slightly improve the object feature extraction ability. Compared with the baseline algorithm, the AI-TOD and DOTA datasets increase the *AP* value by 5.2% and 2.6% respectively. Compared with the original JS divergence formula, the improved geometric JS divergence with closed-form formula can better improve the performance of object detectors, and the *AP* value is increased by 5.9% and 2.2% respectively. We believe that the JSDM module can greatly improve the detection results. This module provides a more accurate anchor box regression calculation method, which alleviates two shortcomings of IoU threshold calculation (i.e., imbalance in the number of positive and negative samples for tiny objects and imbalance in scale samples). Compared to using the original JS divergence formula, geometric JS divergence belongs to a more accurate closed form, which can reduce the systematic error of numerical calculation, and thus obtain better detection results for tiny objects.

**Table 1:** Ablation study on AI-TOD and DOTA datasets.

**Component/dataset**	**Baseline**	**Different setting of JSDNet**				
Swin-trans.		✓			✓	✓
JSDM-Ori.			✓		✓	
JSDM				✓		✓
AI-TOD	24.2	29.4	40.1	46.6	46.3	52.2
DOTA	62.0	64.6	68.5	70.7	70.9	73.1

Table 2 explores the impact of the weight coefficients of geometric JS divergence on detection performance on the AI-TOD dataset. As can be seen, when α = 0.5, the detector was able to achieve the optimal detection effect, with the *AP*_50_ value reaching 52.2%. The smaller or larger the value of α, the more unbalanced the coupling between the covariance matrices of the two Gaussian distributions. This will lead to deviations in the regression constraints, and weakening the detection effect. The experiment shows that the improved geometric JS divergence can obtain closed form calculation results. When the covariance matrices of two Gaussian distributions are balanced coupled together, better detection results can be obtained, and these results are approximately symmetric.

**Table 2:** Effect study of different α values on AI-TOD dataset.

**α**	**α = 0.1**	**α = 0.2**	**α = 0.3**	**α = 0.4**	**α = 0.5**
*AP*_50_	43.2	47.1	49.7	51.6	52.2
α	α = 0.6	α = 0.7	α = 0.8	α = 0.9	
*AP*_50_	51.5	50.4	48.3	45.0	

### 4.3. Comparison and discussion

This section evaluates JSDNet and various algorithms on AI-TOD and DOTA datasets.

(1) AI-TOD dataset: We have conducted experiments on some baseline object detectors, including methods with and without anchor box. Table 3 is a comparison of the quantitative results of the algorithm, listing the *AP* value calculation results for different thresholds and scales. It can be seen that the proposed algorithm significantly improves the detection performance of tiny objects in remote sensing. JSDNet achieved 52.2% on the *AP*_50_ and 13.0% on the *AP*_75_, leading other methods, including the GWD and KLD methods under horizontal bounding box detection. CenterNet and YOLOv5 have achieved good results in traditional detectors, but it is clear that these methods are weak for tiny object detection. Experiment results demonstrate the effectiveness of using the analytic form of geometric JS divergence in the measurement of object detection distribution, and achieve the most advanced performance. The *AP*_*vt*_ and *AP*_*t*_ represent the evaluation of tiny object detection, with JSDNet reaching 8.6% and 19.3%, respectively, which is better than other methods, indicating that JSDNet can effectively learn the geometric JS divergence representation of remote sensing tiny objects, thereby avoiding the traditional IoU calculation.

**Table 3:** Comparison of quantitative results of different indicators on AI-TOD.

**Methods**	**Backbone**	***AP***	***AP*_50_**	***AP*_75_**	***AP*_*vt*_**	***AP*_*t*_**	***AP*_*s*_**	***AP*_*m*_**
**Anchor-free**								
PepPonits (Yang et al., 2019)	Resnet-50	9.2	23.6	5.3	2.5	9.2	12.9	14.4
FoveaBox (Kong et al., 2020)	Resnet-50	11.3	28.1	7.4	1.4	8.6	17.8	32.2
FCOS (Tian et al., 2019)	Resnet-50	12.0	30.2	7.3	2.2	11.1	16.6	26.9
Grid R-CNN (Lu et al., 2019)	Resnet-50	14.3	31.1	11.0	0.1	11.0	25.7	36.7
**Two-stage**								
TridentNet (Li et al., 2019)	Resnet-50	10.1	24.5	6.7	0.1	6.3	19.8	31.9
Faster R-CNN (Ren et al., 2017)	Resnet-50	12.8	29.9	9.4	0.0	9.2	24.6	37.0
Cascade R-CNN (Cai and Vasconcelos, 2018)	Resnet-50	15.1	34.2	11.2	0.1	11.5	26.7	38.5
DetectoRS (Qiao et al., 2021)	Resnet-50	16.1	35.5	12.5	0.1	12.6	28.3	**40.0**
**One-stage**								
RetinaNet (Lin et al., 2020)	Resnet-50	8.9	24.2	4.6	2.7	8.4	13.1	20.4
SSD (Liu et al., 2016)	VGG-16	10.7	32.5	4.0	2.0	8.7	16.8	28.0
YOLOv5 (Bochkovskiy et al., 2020)	DarkNet-53	11.5	36.6	4.7	3.5	9.1	19.2	27.2
CenterNet (Duan et al., 2019)	DLA-34	16.7	37.1	3.7	2.8	10.1	15.5	18.0
GWD-hor (Yang et al., 2021a)	Resnet-101	17.0	41.9	7.8	4.4	15.3	22.7	28.8
KLD-hor (Yang et al., 2021b)	Resnet-101	17.7	44.3	11.3	4.8	17.1	23.6	30.3
JSDNet (ours)	Resnet-50	18.2	46.6	10.5	5.4	15.9	24.4	31.6
JSDNet (ours)	Resnet-101	19.8	49.4	11.6	7.3	18.7	26.4	32.4
JSDNet (ours)	Swin-Trans	**21.4**	**52.2**	**13.0**	**8.6**	**19.3**	**29.0**	35.7

Bold values indicate the maximum value of the vertical column.

**Figure 4:**
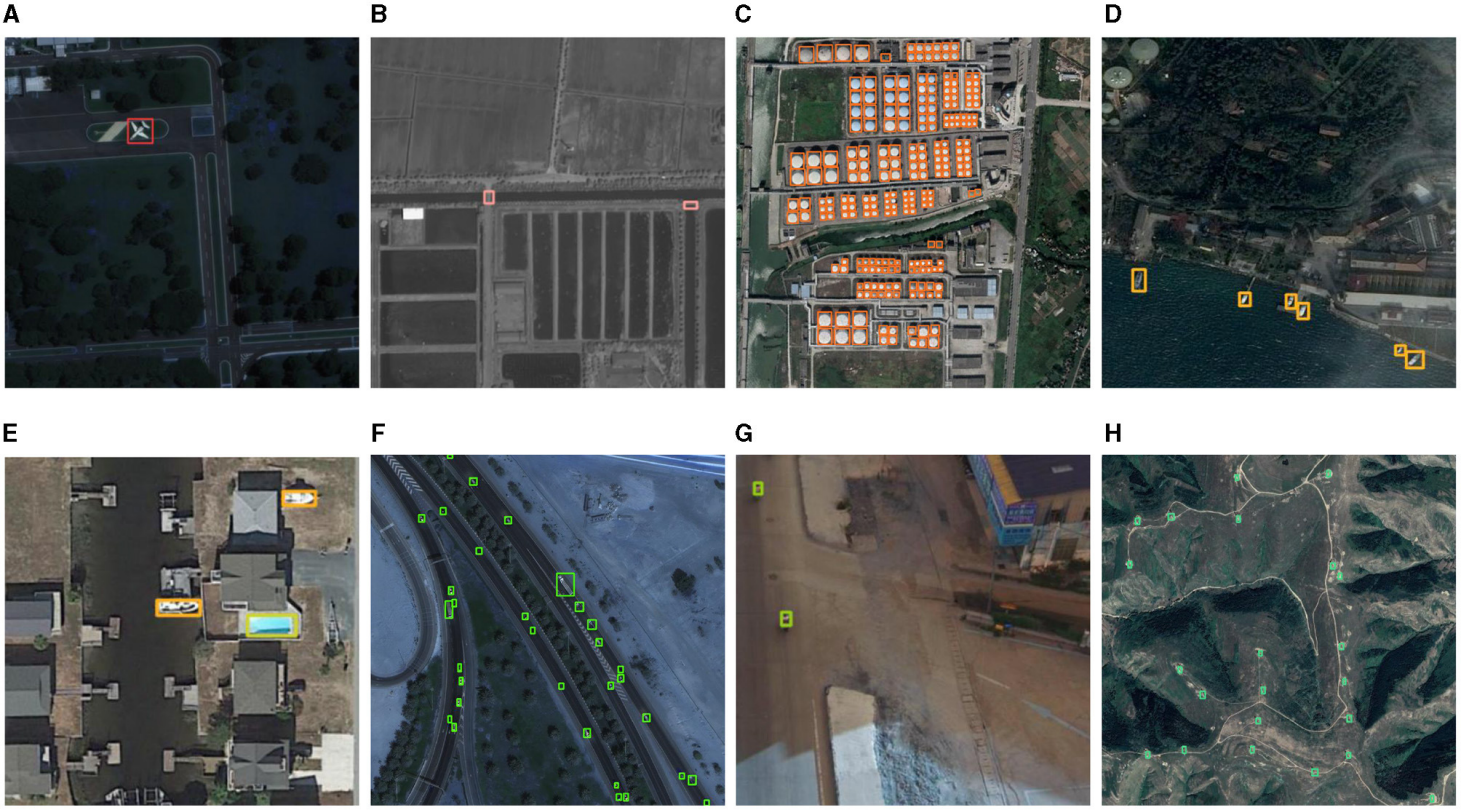
Qualitative inference results of JSDNet on AI-TOD. **(A)** Airplane. **(B)** Bridge. **(C)** storage tank. **(D)** Ship. **(E)** Swimming pool. **(F)** Vehicle. **(G)** Person. **(H)** Wind mill.

Table 4 shows the detection results for eight object categories in the AI-TOD dataset. The proposed method is leading in terms of effectiveness in six categories, only second to the optimal results in the other two categories. The horizontal bounding box detection results using Wasserstein distance and KL divergence for distance measurement are listed in the table. Although they have also achieved good results, they are not closed formulas in information geometry, resulting in errors in the similarity measurements. Therefore, using the geometric JS divergence method achieves better detection performance. In addition, in some challenging object categories, such as SP, PE, WM, etc., JSDNet has advantages in detection effectiveness. The distribution of samples in these categories is uneven, and the background around the object is complex. Therefore, all methods have obtained lower AP values. Figure 4 shows some qualitative reasoning results for JSDNet. It is worth noting that JSDNet can accurately detect densely distributed tiny objects, such as vehicles, ships, storage tanks, and so on. Although JSDNet uses a horizontal bounding box, from the visual effect, the horizontal box is more suitable for positioning tiny objects in remote sensing image, and using a rotation box has little significance.

**Table 4:** Comparison of quantitative results of different categories on AI-TOD.

**Methods**	**Backbone**	**APL**	**BR**	**ST**	**SH**	**SP**	**VE**	**PE**	**WM**	***AP*_50_**
**Anchor-free**										
PepPonits (Yang et al., 2019)	Res-50	0.0	0.1	22.5	28.8	0.2	18.3	4.1	0.0	23.6
FoveaBox (Kong et al., 2020)	Res-50	15.6	3.3	21.1	20.8	9.7	16.3	4.0	0.0	28.1
FCOS (Tian et al., 2019)	Resnet-50	7.2	13.4	20.2	26.7	8.4	16.3	3.5	0.0	30.2
Grid R-CNN (Lu et al., 2019)	Resnet-50	24.5	11.7	20.9	23.5	12.1	16.1	5.1	0.4	31.1
**Two-stage**										
TridentNet (Li et al., 2019)	Resnet-50	19.3	0.1	17.2	16.2	12.4	12.5	3.4	0.0	24.5
Faster R-CNN (Ren et al., 2017)	Resnet-50	19.7	4.8	19.0	19.9	3.7	14.4	4.8	0.0	29.9
Cascade R-CNN (Cai and Vasconcelos, 2018)	Resnet-50	26.2	9.6	24.0	24.3	13.2	17.5	5.8	0.1	34.2
DetectoRS (Qiao et al., 2021)	Resnet-50	28.5	11.7	23.2	26.4	14.9	17.6	6.5	0.2	35.5
**One-stage**										
RetinaNet (Lin et al., 2020)	Resnet-50	1.3	11.8	14.3	23.6	5.8	11.4	2.3	0.5	24.2
SSD (Liu et al., 2016)	VGG-16	14.9	9.6	13.2	18.2	10.6	12.7	2.9	3.1	32.5
YOLOv5 (Bochkovskiy et al., 2020)	DarkNet-53	19.6	10.7	11.3	22.0	9.2	14.3	3.7	0.9	36.6
CenterNet (Duan et al., 2019)	DLA-34	29.2	13.1	22.9	27.7	**15.6**	19.0	7.2	0.2	37.1
GWD-hor (Yang et al., 2021a)	Resnet-101	26.3	12.6	28.1	25.5	13.1	21.3	5.9	3.5	41.9
KLD-hor (Yang et al., 2021b)	Resnet-101	25.1	13.8	28.9	27.4	14.3	22.0	6.2	4.1	44.3
JSDNet (ours)	Resnet-50	25.8	15.8	30.4	29.7	12.5	20.6	6.0	4.9	46.6
JSDNet (ours)	Resnet-101	27.1	**16.4**	33.6	31.5	13.9	23.0	7.2	5.7	49.4
JSDNet (ours)	Swin-Trans	**29.9**	16.2	**34.4**	**33.0**	14.7	**26.5**	**8.6**	**7.9**	**52.2**

Bold values indicate the maximum value of the vertical column.

**Figure 5:**
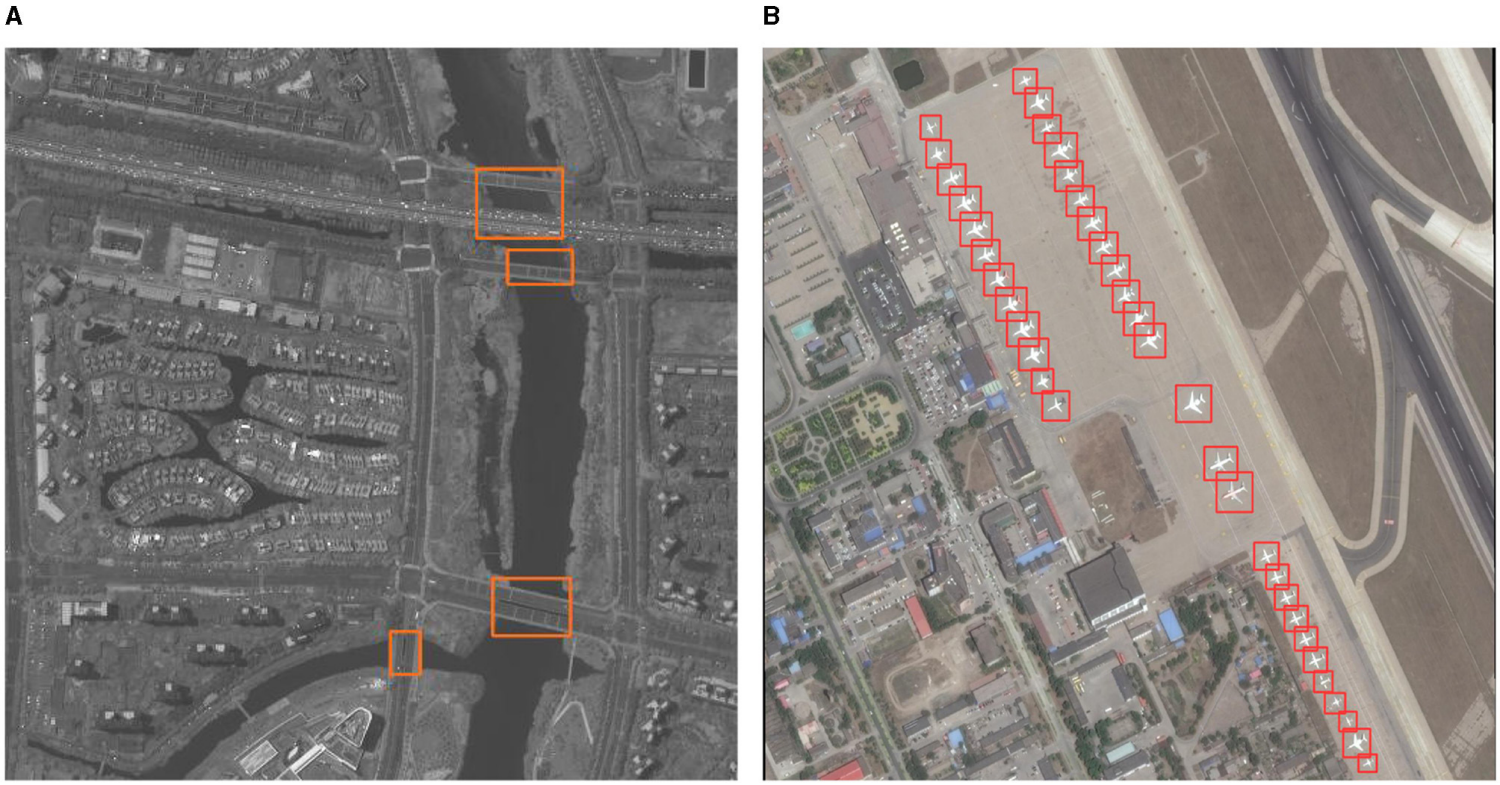
Qualitative inference results of JSDNet on DOTA. **(A)** Bridge. **(B)** Plane.

(2) DOTA dataset: Table 5 lists the detection results of JSDNet and some baseline algorithms on the DOTA dataset. When using Resnet-50 as the backbone network, the detection results of this method are still good, with *AP*_50_ achieving 70.7%. In terms of *AP*_*vt*_ and *AP*_*t*_ indicators, some general detectors performed weakly. We believe that this is due to the impact of IoU calculation and threshold setting for tiny objects, while the GWD, KLD and JSDNet with horizontal bounding box have improved this issue somewhat. When using Swin Transformer as the backbone network, JSDNet can extract features of tiny objects more sufficient, improving the detection results. The *AP*_50_ achieved 73.1%. Figure 5 is visual result of JSDNet on the DOTA test set. JSDNet can accurately regress the spatial location information of tiny objects. The figure shows the detection effect of bridges and airplanes, which belong to smaller objects in the dataset and can still be accurately located.

**Table 5:** Comparison of quantitative results on DOTA.

**Methods**	**Backbone**	***AP***	***AP*_50_**	***AP*_75_**	***AP*_*vt*_**	***AP*_*t*_**	***AP*_*s*_**	***AP*_*m*_**
Faster R-CNN (Ren et al., 2017)	Resnet-50	35.6	59.5	37.2	0.0	7.1	28.9	42.1
Cascade R-CNN (Cai and Vasconcelos, 2018)	Resnet-50	37.0	59.5	39.6	0.0	5.9	28.4	44.0
DetectoRS (Qiao et al., 2021)	Resnet-50	40.8	62.6	44.4	0.0	7.0	29.9	47.8
RetinaNet (Lin et al., 2020)	Resnet-50	40.5	62.0	43.9	0.1	6.5	30.2	46.7
GWD-hor (Yang et al., 2021a)	Resnet-101	41.1	63.8	43.3	0.4	8.5	30.5	48.7
KLD-hor (Yang et al., 2021b)	Resnet-101	41.8	67.2	44.2	0.8	9.4	32.0	50.1
JSDNet (ours)	Resnet-50	43.4	70.7	44.9	1.3	10.1	32.4	50.3
JSDNet (ours)	Swin-Trans	**45.2**	**73.1**	**47.0**	**1.7**	**12.9**	**34.2**	**52.4**

Bold values indicate the maximum value of the vertical column.

## 5. Conclusion

The sensitivity of tiny object detection in remote sensing images to the IoU threshold and the IoU calculation process makes a robust tiny object detector particularly important. A small position offset leads to a large change in the IoU value. Therefore, this article has adopted the closed-form of geometric JS divergence representation of tiny objects as the similarity measure for bounding-box distribution. In this article, the Swin Transformer model is adaptively integrated into the tiny object detection network to efficiently extract tiny features. The JSDM module is based on the Gaussian distribution modeling of the ground-truth and anchor box, and then the geometric JS divergence with the closed-form formula is applied to measure the distribution distance. The ablation and comparison experiments have been carried out on AI-TOD and DOTA datasets, and the results show that the proposed JSDNet can effectively improve the performance of remote sensing tiny object detection and can fully learn the geometric JS divergence representation of tiny objects.

## Data availability statement

The original contributions presented in the study are included in the article/supplementary material, further inquiries can be directed to the corresponding author.

## Author contributions

SN: Conceptualization, Resources, Writing—review & editing. CL: Methodology, Writing—review & editing. HW: Software, Writing—original draft. YLi: Validation, Writing—review & editing. YLia: Formal analysis, Investigation, Writing—review & editing. NL: Writing—review & editing.
